# Exploring the Relationship between Cerebral Palsy and Hip Dysplasia: Insights from the National Inpatient Sample

**DOI:** 10.3390/medicina60091394

**Published:** 2024-08-26

**Authors:** Reem Abdullah Alyoubi, Huda Yahya Alyahyawi, Abrar Nayel Alsharief, Ghadeer Ghazi Alahmadi, Faris Althubaiti, Mazen A. Basheikh, Elham H. Alhifthy, Ahmed Abu-Zaid

**Affiliations:** 1 Department of Pediatrics, King Abdulaziz University Hospital, Jeddah 21589, Saudi Arabia; 2 Department of Medicine, King Abdulaziz University Hospital, Jeddah 21589, Saudi Arabia; 3 Department of Internal Medicine, University of Jeddah, Jeddah 23218, Saudi Arabia; 4 Department of Pediatrics, College of Medicine, Princess Nourah bint Abdulrahman University, Riyadh 11671, Saudi Arabia; 5 Department of Biochemistry and Molecular Medicine, College of Medicine, Alfaisal University, Riyadh 11533, Saudi Arabia

**Keywords:** cerebral palsy, hip dysplasia, National Inpatient Sample, prevalence, risk factors

## Abstract

*Background and Objective*: Cerebral palsy (CP) significantly impacts quality of life globally. Hip dysplasia (HD) is a common musculoskeletal issue in CP patients. This study investigates the prevalence, risk factors, and impact of HD on CP patients using a large national database. *Materials and Methods*: Data from the National Inpatient Sample (NIS) database (2016–2019) were used, identifying CP and HD diagnoses through ICD-10 codes. Baseline characteristics were tabulated. Univariate and multivariate logistic regression analyses examined predictors of HD development in CP patients, presenting data as odds ratios (ORs) with 95% confidence intervals (CIs). *Results*: Among 3,951,040 pediatric patients, 28,880 had CP (27,466 without HD, and 1414 with HD), and 3,922,160 did not have CP. CP significantly increased the likelihood of developing HD in univariate (OR = 35.03, 95% CI [33.01, 37.17], *p* < 0.0001) and multivariate (OR = 26.61, 95% CI [24.94, 28.40], *p* < 0.0001) analyses. Among patients with CP, race was significantly associated with HD, with ORs below 1 for all racial categories compared to Whites. Females had nearly twice the odds of HD compared to males (OR = 1.96, 95% CI [1.86, 2.05], *p* < 0.0001). Age was significantly associated with HD, with each additional year increasing the odds (OR = 1.03, 95% CI [1.026, 1.034], *p* < 0.0001). Individuals in the high 51st–75th income quartile had a 17% increase in the odds of HD (OR = 1.17, 95% CI [1.09, 1.25], *p* < 0.0001) compared to the low 1st–25th income quartile. *Conclusions*: This study reinforces the strong association between CP and HD, highlighting the need for further research and prospective studies to validate these findings.

## 1. Introduction

Cerebral palsy (CP) describes a wide range of neurological impairments arising from injury or abnormal development of the immature brain [1,2]. This results in many motor dysfunctions, including spasticity, dyskinesia, ataxia, and hypertonia, which manifest as disturbances in movement, muscle tone, coordination, and posture [3]. CP significantly impacts various aspects of an individual’s life, encompassing social, cognitive, communicative, and emotional domains, thereby exerting a profound influence on overall quality of life [4]. In fact, CP is credited as the predominant cause of childhood physical disability worldwide, impacting an estimated two to three children per one thousand live births [1,5].

Hip dysplasia (HD) stands out as a significant complication exacerbating the burden and diminishing the quality of life in CP patients [1,6]. It is considered the second most common musculoskeletal defect in CP patients, preceded only by defects of the foot and ankle [1]. HD is linked to the severity of neurological, functional impairment and the type of CP [6,7]. Approximately one-third of children with CP experience HD, with a markedly higher prevalence of 82% observed among those with spastic quadriplegia compared to other CP subtypes [6]. Moreover, the hazard of developing HD increases with the severity of CP [1,6,8].

HD arises when the hip joint fails to develop correctly, leading to instability and misalignment [8]. It is commonly characterized by an improperly formed hip acetabulum that may be too shallow or poorly positioned, causing the femoral head to not fit securely within the joint [8]. Neuromuscular abnormalities in CP patients such as spasticity can cause involuntary muscle contractions and stiffness [9]. These abnormal muscle contractions can exert additional forces on the hip joint, leading to increased stress and instability. As a result, the already compromised hip joint in individuals with HD may experience further malalignment and deterioration [8]. This condition can result in significant morbidity and decreased quality of life, if left untreated [6]. The progression of HD can lead to dislocation, having serious consequences like intense pain, severe contractures, pelvic obliquity, and scoliosis, leading to disabilities in sitting, standing, and walking [1,10,11]. 

This study aims to explore the link between CP and HD, with a focus on identifying the risk factors associated with the development of HD in pediatric patients using a large population-based study from the National Inpatient Sample (NIS) from 2016 to 2019. This dataset represents the largest sample size when compared to other published studies focusing on CP and HD. We aim to fill the possible gap in the literature with robust large analysis and avoid weak conclusions due to small sample size as in previous studies. Through analysis and examination of relevant data, the findings could have far-reaching implications for healthcare strategies, which will aid in formulating methods to prevent and manage HD in children with CP.

## 2. Materials and Methods

### 2.1. Ethical Approval

Due to the publicly available nature of the NIS database and de-identified data, ethical approval was not required.

### 2.2. Study Design

We conducted a retrospective cohort study to investigate the association between CP and HD, aiming to identify the risk factors linked to the development of HD in pediatric patients through analysis of a large population-based dataset. Our dataset was sourced from the NIS covering the period from 2016 to 2019. The NIS is an extensive database offered by the Agency for Healthcare Research and Quality in the United States. It is the largest publicly available database of inpatient hospital stays in the United States. It provides comprehensive data on patient demographics, diagnoses, treatments, and outcomes from a broad range of hospitals across the country. It is designed to support research and policy analysis on hospital care and health trends, offering a valuable resource for understanding patterns in hospital utilization and patient outcomes [12]. 

### 2.3. Eligibility Criteria, Participants, and Sample Size

The inclusion criteria for our study were pediatric patients aged 18 years or younger with complete baseline characteristic information. We excluded non-pediatric patients, as well as pediatric patients with incomplete baseline data. To identify patients with CP and HD, we used the International Classification of Diseases, 10th Edition (ICD-10) codes. The specific ICD-10 codes for CP, HD, and baseline characteristics are detailed in Table 1. Patient classification into CP with and without HD, and non-CP with and without HD, was based solely on the availability of relevant ICD-10 codes. After applying these criteria, we excluded 1,641,602 patients with missing data and 22,891,445 patients over 18 years old. Of the remaining 3,951,040 eligible admissions, we identified 28,880 patients with CP (27,466 without HD and 1414 with HD) and 3,922,160 patients without CP (3,916,404 without HD and 5756 with HD).

### 2.4. Data Collection and Statistical Analysis

In the descriptive analysis, we examined baseline characteristics, including age, sex, race, primary payer, median household income (categorized into quartiles), low birth weight, preterm, hospital status, hospital bed size, and hospital region. Numerical data were presented with accompanying percentages, while age was conveyed as mean and standard deviation. The Chi square test (x^2^) was used to assess the statistical significance of difference across the CP with versus without HD for categorical variables. Additionally, we employed inferential statistics, utilizing univariate and multivariate regression analyses to investigate the role of CP in the development of HD among the pediatric population. The multivariate regression model incorporated adjustments for covariates, including factors such as race, female gender, age, hospital bed size, hospital location, hospital region, primary payer, low birth weight, and preterm delivery (Table 1). Data were summarized as odds ratios (ORs) with corresponding 95% confidence intervals (CIs). Statistical significance was declared at *p* value < 0.05. The statistical analysis was completed using the Stata software (version 15, StataCorp LLC, College Station, TX, USA).

## 3. Results 

### 3.1. Baseline Characteristics

Table 2 offers a detailed analysis of demographic and clinical characteristics in 28,880 CP individuals with (n = 1414) and without (n = 27,466) HD. The mean age of those with HD was slightly lower at 9.27 years, compared to 9.41 years in those without HD. Gender distribution showed a minor difference, with 59.34% males among those with HD and 57.53% in those without HD. Notably, there was a statistically significant difference in the primary expected payer category (*p* < 0.001), indicating a higher prevalence of private insurance (33.45%) among individuals with HD compared to those without HD (28.33%). Although race distribution did not show an overall significant difference, there was a slight variation among different racial groups. Variables such as year of admission, ZIP code income quartile, hospital bed size, hospital status, region, preterm, and low birth weight categories exhibited similar proportions for CP individuals with and without HD. This comprehensive comparison provides nuanced insights into the demographic and clinical characteristics associated with the presence of HD in this cohort of CP patients.

### 3.2. Association between CP and HD

Table 3 offers a detailed analysis of factors influencing HD development in pediatric patients. Notably, CP had a substantial impact, with statistical significance, on the likelihood of developing HD in the univariate (OR = 35.03, 95% CI [33.01, 37.17], *p* < 0.0001) and multivariate (OR = 26.61, 95% CI [24.94, 28.40], *p* < 0.0001) analyses. Additionally, in multivariate analyses, race showed significant associations with HD, with ORs below 1 for all racial categories compared to the reference group (White), indicating a lower likelihood of HD in these groups. In addition to this, female gender showed nearly twice the odds of HD compared to males (OR = 1.96, 95% CI [1.86, 2.05], *p* < 0.0001). Age also exhibited a significant association, with each incremental year correlating with a marginal increase in the odds of HD (OR = 1.03, 95% CI [1.026, 1.034], *p* < 0.0001), suggesting a progressive nature of HD with advancing age in CP patients. ZIP code income quartiles indicated that higher income levels were associated with a greater likelihood of HD. Specifically, individuals in the 51st–75th income quartile had a 17% increase in the odds of HD (OR = 1.17, 95% CI [1.09, 1.25], *p* < 0.0001) compared to the reference group (1st–25th income quartile). Similarly, those in the 76th–100th income quartile exhibited even higher odds of 27% of developing HD (OR = 1.27, 95% CI [1.19, 1.36], *p* < 0.0001).

Hospital characteristics, encompassing bed size and teaching status, revealed noteworthy correlations. Specifically, receiving treatment in urban teaching hospitals was linked to markedly increased odds of HD (OR = 2.79, 95% CI [2.4, 3.23] *p* < 0.0001), suggesting potential disparities in treatment approaches or patient populations (Table 3).

## 4. Discussion 

The relation between CP and HD presents a substantial challenge, imposing a considerable burden on individuals affected by both conditions [1,2]. The recent literature underscores the heightened vulnerability of individuals with CP to the development of HD, a condition characterized by abnormal hip joint development [6,13]. CP alone poses challenges in motor coordination and muscle control, while the addition of HD exacerbates functional limitations and discomfort [1]. This relation between CP and HD necessitates a comprehensive understanding of the complex relation between neurological and musculoskeletal aspects. 

The pathophysiology of HD in CP patients is a complex interplay of factors rooted in the motor and musculoskeletal challenges inherent to CP [6]. CP is characterized by impaired motor function and muscle coordination introducing a cascade of challenges that significantly impacts the musculoskeletal system [14]. One key aspect is altered muscle tone and spasticity, which are common traits in CP patients [15]. These neuromuscular abnormalities create an imbalance in the forces exerted on the hip joint, initiating a spectrum of manifestations related to HD [8]. The heightened muscle tone and spasticity experienced by individuals with CP can lead to muscle contractures, which happens in spastic CP [1,9]. This, in turn, affects the alignment of the femoral head within the acetabulum of the hip joint [15]. The resulting misalignment and increased mechanical stresses contribute to the development of HD over time [8]. Furthermore, the altered gait patterns and limited mobility in CP exacerbate the stress on the hip joint, which happens in ataxic CP [16,17]. Also involuntary movements that can be found in dyskinetic CP may affect the stability of the hip joint [17]. These abnormal biomechanical forces, compounded by the neuromuscular issues, impose additional stress on the developing hip joint, potentially hindering its normal growth and maturation [15,17]. The severity of CP is considered the most crucial factor associated with HD, with spastic hemiplegia showing an 83% incidence rate, while the prevalence is comparatively lower in other types [18]. Spastic CP is the most common type of CP, accounting for around 80% of CP cases [19,20]. Also, it is the most common type associated with HD according to several studies [1,21].

Findings from our results showed a strong association between CP and HD in both univariate and multivariable regression analyses. The incidence rate of HD in CP patients in our study was approximately 4.89%, which was lower than previously reported rates of around 35% [6,22]. This can be largely attributed to the limited nature of the NIS database, which considers only inpatient cohorts and excludes outpatient cohorts.

Our analysis revealed that the 76th–100th income quartile exhibited 27% higher odds of HD compared to the reference group (1st–25th income quartile). This suggests a socioeconomic disparity where higher income levels are associated with a heightened risk of HD in CP patients. In our study, among patients with CP, females exhibited nearly twice the odds of developing HD compared to males. This observation aligns with the published literature, which highlights the female gender as a significant risk factor for developing HD [23,24], despite CP being more common in males [25]. 

To our understanding, there are currently no specific drug therapies designed to prevent HD in patients with CP. Instead, a multidisciplinary approach involving hip surveillance, physical therapy, orthotics, and possibly surgical interventions remains the primary method to manage and prevent HD in patients with CP [13]. 

In low socioeconomic countries, combining community-based initiatives, educational programs, and leveraging technology can enhance the surveillance and early detection of HD in individuals with CP [6]. This approach aims to improve the overall well-being and outcomes for these patients. Based on recommendations from numerous articles, we strongly recommend the establishment of a comprehensive national-level hip surveillance program [6]. Empirical evidence from cross-sectional and population-based studies consistently demonstrates that a well-run hip surveillance program can effectively reduce or potentially eliminate the incidence of late-presenting hip dislocation and its associated complications among patients with CP [6,26,27].

In a study published by Howard et al. in 2023, it was found that approximately 20% of children with CP experience premature mortality by the age of 4 years, even in the absence of prior surgical intervention [13]. However, the decision to refrain from surgical intervention for children with severe CP and HD raises ethical concerns, given the difficulty in predicting which children would be at a higher risk of mortality following hip surgery [13]. Additionally, the complication rate after hip surgery in non-ambulant children is significant, with a reported complication rate of 54% when including failure to cure as part of the complications [13]. 

Our study, based on the NIS, benefits from its wide-ranging coverage of diverse patients across the United States and enhances the generalizability of our findings to a broader population, contributing to the robustness of our conclusions. Additionally, the dataset’s longitudinal nature enables us to examine trends over time. However, limitations include potential coding errors, the lack of outpatient data, as NIS captures only inpatient data, and the observational nature of this study, which precludes establishing causation. Also, we could not evaluate the hip surveillance and rehabilitation measures taken by CP and non-CP patients to prevent and treat HD. Nonetheless, our findings offer valuable insights into healthcare outcomes, guiding future research and policy initiatives.

## 5. Conclusions 

Our study strengthens the association between CP and HD. CP significantly increased HD likelihood in both univariate and multivariate analyses. In addition, female gender, older age, and higher income were associated with higher HD odds. Also, race showed lower HD likelihood for non-Whites with CP.

## Figures and Tables

**Table 1:** The ICD-10 codes and NIS demographics used in the analysis.

Variable	Source	ICD-10 Code
Hip dysplasia	I10_DX1/40	Q65.89
Cerebral palsy	I10_DX1/40	G80
Extremely preterm (less than 28 weeks)	I10_DX1/40	P.072
Very preterm (28 to <32 weeks)	I10_DX1/40	P07.31, P0.732, P07.33, P07.34
Moderate-to-late preterm (32 to <37 weeks)	I10_DX1/40	P07.35, P07.36, P07.37, P07.38, P07.39
Extremely low birth weight (<1000 g)	I10_DX1/40	P0.70
Very low birth weight (1000–1499 g)	I10_DX1/40	P07.14, P07.15
Moderately low birth weight (1500–2499 g)	I10_DX1/40	P07.16, P07.17, P07.18
Age	NIS Core/Hospital	-
Female gender	NIS Core/Hospital	-
Primary expected payer	NIS Core/Hospital	-
Race	NIS Core/Hospital	-
Year	NIS Core/Hospital	-
ZIP income quartile	NIS Core/Hospital	-
Location/teaching hospital status	NIS Core/Hospital	-
Hospital bed size	NIS Core/Hospital	-
Length of hospital stay	NIS Core/Hospital	-
In-hospital mortality	NIS Core/Hospital	-

**Table 2:** Summary of demographic and clinical characteristics in CP individuals with and without HD.

Variables	Cerebral Palsy without Hip Dysplasia (n = 27,466)	Cerebral Palsy with Hip Dysplasia (n = 1414)	*p*-Value
**Age (mean ± SD)**	9.41 ± 5.18	9.27 ± 4.24	<0.0001
**Hospital stay (mean ± SD)**	6.55 ± 12.18	5.63 ± 11.21	<0.0001
**In-hospital mortality**	265 (0.96%)	5 (0.35%)	<0.0001
**Sex**			0.179
Male	15,800 (57.53%)	839 (59.34%)	
Female	11,666 (42.47%)	575 (40.66%)	
**Primary expected payer**			0.001
Medicare	89 (0.32%)	4 (0.28%)	
Medicaid	17,737 (64.58%)	828 (58.56%)	
Private insurance	7781 (28.33%)	473 (33.45%)	
Self-pay	223 (0.81%)	13 (0.92%)	
No charge	12 (0.04%)	1 (0.07%)	
Others	1624 (5.91%)	95 (6.72%)	
**Race**			0.115
White	13,140 (47.84%)	697 (49.29%)	
Black	5340 (19.44%)	252 (17.82%)	
Hispanic	6473 (23.57%)	328 (23.20%)	
Asian or Pacific Islander	963 (3.51%)	43 (3.04%)	
Native American	227 (0.83%)	8 (0.57%)	
Other	1323 (4.82%)	86 (6.08%)	
**Calendar year**			0.209
2016	6180 (22.50%)	337 (23.83%)	
2017	6918 (25.19%)	340 (24.05%)	
2018	7178 (26.13%)	345 (24.40%)	
2019	7190 (26.18%)	392 (27.72%)	
**ZIP income quartile**			0.071
1st–25th	8257 (30.06%)	379 (26.80%)	
26th–50th	7395 (26.92%)	391 (27.65%)	
51st–75th	6589 (23.99%)	360 (25.46%)	
76th–100th	5225 (19.02%)	284 (20.08%)	
**Bed size of hospital**			0.253
Small	5616 (20.45%)	301 (21.29%)	
Medium	5495 (20.01%)	258 (18.25%)	
Large	16,355 (59.55%)	855 (60.47%)	
**Hospital status**			<0.001
Rural	333 (1.21%)	12 (0.85%)	
Urban nonteaching	1052 (3.83%)	18 (1.27%)	
Urban teaching	26,081 (94.96%)	1384 (97.88%)	
**Hospital region**			0.430
Northeast	4201 (15.30%)	212 (14.99%)	
Midwest or North Central	6668 (24.28%)	366 (25.88%)	
South	9980 (36.34%)	489 (34.58%)	
West	6617 (24.09%)	347 (24.54%)	
**Preterm**			0.587
Extremely preterm (less than 28 weeks)	96 (0.35%)	7 (0.50%)	
Very preterm (28 to <32 weeks)	145 (0.53%)	5 (0.35%)	
Moderate-to-late preterm (32 to 37 weeks)	59 (0.21%)	2 (0.14%)	
**Low birth weight**			0.606
Extremely low birth weight (less than 1 kg)	15 (0.05%)	0 (0.00%)	
Very low birth weight (1 to 1.5 kg)	14 (0.05%)	0 (0.00%)	
Moderately low birth weight	35 (0.13%)	1 (0.07%)	

**Table 3:** Summary of multivariate regression analysis of factors influencing HD development in pediatric patients.

Variables	Odds Ratio	Lower 95% CI	Upper 95% CI	*p* Value
**Cerebral palsy**	26.61	24.94	28.40	<0.0001
**Race**				
Black	0.51	0.47	0.55	<0.0001
Hispanic	0.88	0.83	0.94	<0.0001
Asian or Pacific Islander	0.69	0.61	0.78	<0.0001
Native American	0.57	0.41	0.79	0.001
Other	0.86	0.78	0.95	0.003
**Female gender**	1.96	1.86	2.05	<0.0001
**Age**	1.03	1.026	1.034	<0.0001
**ZIP income quartile**				
26th–50th	1.09	1.02	1.16	0.011
51st–75th	1.17	1.09	1.25	<0.0001
76th–100th	1.27	1.19	1.36	<0.0001
**Hospital bed size**				
Medium	0.79	0.73	0.85	<0.0001
Large	1.04	0.98	1.10	0.212
**Hospital status**				
Urban nonteaching	1.11	0.94	1.32	0.216
Urban teaching	2.79	2.40	3.23	<0.0001
**Hospital region**				
Midwest or North Central	1.15	1.06	1.24	<0.0001
South	0.99	0.92	1.06	0.722
West	1.09	1.02	1.18	0.018
**Preterm**				
Late preterm birth	1.10	0.95	1.27	0.199
Extremely preterm	0.76	0.51	1.11	0.152
Very preterm	0.93	0.63	1.38	0.722
Moderate-to-late preterm	0.90	0.29	2.81	0.851
**Low birth weight**				
Extremely low birth weight	0.86	0.69	1.08	0.192
Very low birth weight	1.40	0.92	2.14	0.118
Moderately low birth weight	0.74	0.41	1.32	0.310

Abbreviations—CI: confidence interval.

## Data Availability

Data were obtained from a publicly available database under the Hospital Cost and Utilization Project’s Nationwide Inpatient Sample, which can be accessed here: https://www.hcup-us.ahrq.gov/db/nation/nis/nisdbdocumentation.jsp (accessed on 10 July 2024).
